# Cross-Sectional and Longitudinal Hippocampal Atrophy, Not Cortical Thinning, Occurs in Amyloid-Negative, p-Tau-Positive, Older Adults With Non-Amyloid Pathology and Mild Cognitive Impairment

**DOI:** 10.3389/fnimg.2022.828767

**Published:** 2022-06-02

**Authors:** Swati Rane Levendovszky

**Affiliations:** Department of Radiology, School of Medicine, University of Washington, Seattle, WA, United States

**Keywords:** cortical thickness, A/T/N, amyloid, ptau, mild cognitive impairment (MCI)

## Abstract

**Introduction:**

Alzheimer's disease (AD) is a degenerative disease characterized by pathological accumulation of amyloid and phosphorylated tau. Typically, the early stage of AD, also called mild cognitive impairment (MCI), shows amyloid pathology. A small but significant number of individuals with MCI do not exhibit amyloid pathology but have elevated phosphorylated tau levels (A-T+ MCI). We used CSF amyloid and phosphorylated tau to identify the individuals with A+T+ and A-T+ MCI as well as cognitively normal (A-T-) controls. To increase the sample size, we leveraged the Global Alzheimer's Association Interactive Network and identified 137 MCI+ and 61 A-T+ MCI participants. We compared baseline and longitudinal, hippocampal, and cortical atrophy between groups.

**Methods:**

We applied ComBat harmonization to minimize site-related variability and used FreeSurfer for all measurements.

**Results:**

Harmonization reduced unwanted variability in cortical thickness by 3.4% and in hippocampal volume measurement by 10.3%. Cross-sectionally, widespread cortical thinning with age was seen in the A+T+ and A-T+ MCI groups (*p* < 0.0005). A decrease in the hippocampal volume with age was faster in both groups (*p* < 0.05) than in the controls. Longitudinally also, hippocampal atrophy rates were significant (*p* < 0.05) when compared with the controls. No longitudinal cortical thinning was observed in A-T+ MCI group.

**Discussion:**

A-T+ MCI participants showed similar baseline cortical thickness patterns with aging and longitudinal hippocampal atrophy rates as participants with A+T+ MCI, but did not show longitudinal cortical atrophy signature.

## Introduction

Amyloid negative individuals with mild cognitive impairment (MCI) constitute 7–35% of older adults at the risk of Alzheimer's disease (AD) (Wisse et al., 2015). With the new A/T/N framework, such MCI individuals can show low amyloid accumulation, i.e., A-T-N+, A-T+N+, and A-T+N-. This is in contrast with the A+T+ individuals with MCI due to AD pathology who have a high amyloid burden (Jack et al., 2016b, 2018; Dani et al., 2017). Previous studies used hippocampal atrophy as the marker for neurodegeneration, (Caroli et al., 2015; Wisse et al., 2015) and some amyloid-negative MCI cases (also called suspected non-amyloid pathology or SNAP) were likely cases of hippocampal sclerosis (Botha et al., 2018; Jicha and Nelson, 2019), Lewy-body disease, cerebrovascular disease, primary age-related tauopathy (PART), or other degenerative diseases (Abner et al., 2017). Thus, the amyloid negative MCI cohort is heterogeneous and not clearly defined.

Besides amyloid, tau accumulation, especially phosphorylated tau (p-tau), is an essential contributor to AD pathology and cognitive symptoms. Abnormally hyperphosphorylated tau occurs in AD and related degenerative diseases such as frontotemporal dementia, corticobasal degeneration, and progressive supranuclear palsy, as well as in vascular dementia and Lewy Body disease (Hampel et al., 2010). However, the tau variants are different in different disorders. Levels of p-tau_181_ distinguish AD from other degenerative diseases (Mollenhauer et al., 2006). A combination of CSF levels of amyloid and p-tau_181_, therefore improves our specificity to AD (Parnetti et al., 2001; Blennow, 2017). Now, amyloid and p-tau are both considered equally critical with an amyloid-first or a p-tau-first emergence of AD pathology (Small and Duff, 2008; Paul de Paula et al., 2009; Chételat, 2013). Therefore, in this work, we use CSF levels of amyloid and p-tau_181_ (henceforth referred to as p-tau) to identify A-T+ MCI groups with p-tau positivity. The A-T+ MCI group has never been studied before.

The low to modest prevalence of the A-T+ MCI participants makes it difficult to cross-sectionally compare most imaging biomarkers, including cortical thickness (Dani et al., 2017). Therefore, we leverage the Global Alzheimer's Association Interactive Network (GAAIN, http://www.gaain.org/) platform to assemble anatomical imaging data from multiple repositories across the world (Toga et al., 2016). We leverage data harmonization approaches to minimize site-related differences and compare cortical thickness differences and longitudinal rates of atrophy in A-T+ participants, typical participants with MCI due to AD pathology with amyloid positivity (A+T+ MCI) and cognitively normal A-T- controls, i.e., those free of amyloid and p-tau pathology (Fortin et al., 2017).

## Methods

Data used in the preparation of this article were obtained from the Alzheimer's Disease Neuroimaging Initiative (ADNI) database (adni.loni.usc.edu). As such, the investigators within the ADNI contributed to the design and implementation of ADNI and/or provided data but did not participate in the analysis or writing of this report. A complete listing of ADNI investigators can be found at: http://adni.loni.usc.edu/wp-content/uploads/how_to_apply/ADNI_Acknowledgement_List.pdf. Data used in the preparation of this article were obtained from the Alzheimer's Disease Repository Without Borders (ARWiBo – www.arwibo.it). ARWiBo is publicly accessible via neuGRID platform (http://www.neugrid2.eu). A complete listing of ARWiBo researchers can be found at: www.arwibo.it/pdf/ ACKNOWLEDGEMENT.pdf. The EDSD and PharmaCOG (alias E-ADNI) data used in the preparation of this article were obtained from neuGRID platform as well.

### Subject Selection

Using the GAAIN platform, we queried Alzheimer's Disease Neuroimaging Initiative (ADNI) (Jack et al., 2008), European Diffusion Tensor Imaging Study on Dementia (EDSD) (Brueggen et al., 2016), Alzheimer's Disease Repository Without Borders (ARWiBo) (Frisoni et al., 2009), and Prediction of cognitive properties of new drug candidates for neurodegenerative diseases in early clinical development (PharmaCog) (Galluzzi et al., 2016). CSF analyses were included only from a single run to minimize batch effects. This also limits the number of participants available for analysis. Only A-T- controls (NC) and participants with A+T+ MCI and A-T+ MCI were selected from each repository based on CSF amyloid and p-tau. For ADNI, the CSF cut-offs for amyloid, i.e., Aβ_42_ and p-tau were 192 pg/ml and 22 pg/ml, respectively (Shaw et al., 2009). For ARWiBo, the corresponding cut-offs were 687 pg/ml and 61 pg/ml, respectively. EDSD and PharmaCog provided binarized variables for amyloid and p-tau positivity. In the A+T+ MCI group 57% were N+ and 55% of A-T MCI groups were N+. N+ status was based on a cut-off of 93 pg/ml in ADNI and 492 pg/ml in ARWiBo. EDSD and PharmaCog provided binarized variables for tau positivity. Thus, the distribution of N+ and N- in our MCI groups was very similar and would not bias the observed atrophy patterns in one group over the other. We did not consider the status of N, since the N marker is not specific to AD.

We identified a total of 20 A-T- controls (NC), 172 A+T+ MCI, and 92 A-T+ MCI participants for the baseline comparisons and 18 A-T- controls (NC), 137 A+T+ MCI, and 61 A-T+ MCI participants for the longitudinal comparisons (Supplementary Table 1). ADNI only included NC participants who had imaging and CSF samples. Furthermore, of the 115 total NC participants identified in ADNI with imaging and CSF at baseline, only 22 NC participants included in this study were identified as A-T-, 35 were A-T+, and the rest were A+. Longitudinal data was only available in ADNI and PharmaCog. Their average Mini-Mental State Examination (MMSE) scores were 29±1 (Folstein et al., 1983). The NC participants were 75.3 ± 6.5 years old and included 9 males. The 172 A+T+ MCI participants were 73.5 ± 7.5 years old and included 88 males. Average MMSE scores for A+T+ MCI participants were 26± 2. The 92 A-T+ MCI participants were 71.5 ± 7.3 years old and included 47 males. Average MMSE scores were 27 ± 2. The MMSE scores in A+T+ MCI groups were significantly lower (*p* < 0.001) from the A-T+ MCI group and the control group. Although, numerically lower MMSE scores were observed in the A-T+ MCI group compared to the control group, the differences were not significant. All participants were scanned on 3T MRI scanners at different sites with 41 different scanners using standard 3D T1-weighted MRI sequences. Imaging parameters were as outlined in Jack et al. (2008), Frisoni et al. (2009), Teipel et al. (2012), Brueggen et al. (2016), and Galluzzi et al. (2016).

### Baseline Cortical Thickness and Hippocampal Measurements

We applied the standard FreeSurfer (v6.0) pipeline comprising intensity normalization, skull tissue removal, tissue segmentation, resampling to a 1 × 1 × 1 mm (Jack et al., 2016a) resolution, and registration with the standard FreeSurfer brain (fsaverage) (Fischl, 2012). Briefly, this pipeline removes non-brain tissue, performs automated Talairach transformation, segments subcortical white matter and deep gray matter volumetric structures, performs intensity normalization and tessellation of the gray/white matter boundary, and detects the gray/white and gray/cerebrospinal fluid borders. No manual editing was performed. We randomly sampled 10% of the data and visually inspected them to ensure accurate segmentation.

Next, we performed a generalized linear model (GLM) analysis to evaluate group differences between NC, A+T+ MCI, and A-T+ MCI participants. Since the sample size for NC is sub-optimal, we did not expect significant differences between amyloid positive MCI and NC and between A-T+ MCI and NC groups. All comparisons were adjusted for age and gender. We also evaluated the relationship between cortical thickness and age within each group and whether this relationship was different between groups. This comparison was adjusted for gender. Analysis was performed using standard FreeSurfer pipeline on a vertex-by-vertex basis in surface space, followed by cluster-wise correction for multiple comparison using 5,000 permutations. All data were registered to the standard “fsaverage” space in FreeSurfer. Significance at the vertex level was considered at *p* = 0.0001 using a two-sided *t*-test. The cluster-wise *p*-value was 0.05, i.e., the probability of seeing that cluster during the 5,000 simulations. Details of this approach are outlined in (Hagler et al., 2006).

### Harmonization of Multi-Site Baseline Data

Using the ComBat approach with parametric adjustments, we harmonized baseline cortical thickness measurements to minimize site effects (Fortin et al., 2017). ComBat uses an empirical Bayes approach to estimate additive and multiplicative effects of scanner-related systematic variations in the cortical thickness values. Biological variability is preserved by providing relevant parameters such as cognitive status, age, and gender. ComBat has been used effectively in many neuroimaging studies to harmonize data across sites (Fortin et al., 2017, 2018). The aforementioned analyses were repeated after harmonization, and the differences in the outcomes were evaluated. If a site had multiple scanners, then each scanner was assigned as a separate site for harmonization purposes. The coefficient of variation was measured at each vertex (ratio of standard deviation across all participants to the average value of thickness across all participants) and compared before and after harmonization to ascertain a reduction in systematic variability. Similarly, a regression analysis was performed to determine if the hippocampal volume was associated with the diagnostic group after adjusting for age, gender, and intracranial volume using the “rms” package in R. Coefficient of variation was estimated for hippocampal volume and intracranial volume and compared before and after harmonization. Due to the inclusion of categorical variables (for group) in our model, the linear regression was implemented as an analysis of variance (ANOVA).

### Longitudinal Cortical Thickness and Hippocampal Measurements

Eighteen NC, 137 MCI+, and 61 A-T+ participants were available with longitudinal data. We applied FreeSurfer's longitudinal pipeline to estimate the rates of cortical atrophy (change in thickness per year) (Reuter et al., 2010, 2012; Reuter and Fischl, 2011). All analyses were completed using FreeSurfer's standard longitudinal pipeline on a voxel-wise basis followed by a multiple comparison's correction using False Discovery Rate (FDR) of <0.05. We applied a linear mixed-effects model to assess the difference in rates of atrophy between NC and A-T+ MCI groups. Using the longitudinal approach is advantageous because it does not include variability due to sites or scanners. All comparisons were adjusted for age and gender. Finally, the *p*-value cut-offs for the mass-univariate regression equation provided by FreeSurfer were projected on the standard 2D-surface space, i.e., on the “fsaverage” brain. With the small number of NC participants, we did not expect significant differences from comparisons with or within the NC group.

We would like to note that association with cognitive assessments was not a focus of this study. Furthermore, not all studies conduct identical tests or in an identical manner. For instance, ADNI sites use the cognitive battery from the Uniform Data Set (UDS), (Weintraub et al., 2018) while EDSD uses the Consortium to Establish a Registry of Alzheimer's Disease (CERAD) testing battery (Petersen, 2004). This would make the above question difficult to assess correctly. The ARWiBo and PharmaCog studies used a variety of cognitive tests to assess short-term and long-term memory impairment as well as other assessments (for depression, behavior, etc.) to determine MCI status. Due to subjectivity in the selection process and the heterogeneity in the assessment of MCI status, it would be difficult to combine all data and test associations with cognitive performance.

## Results

### Cross-Sectional Comparisons

**Figure 1:**
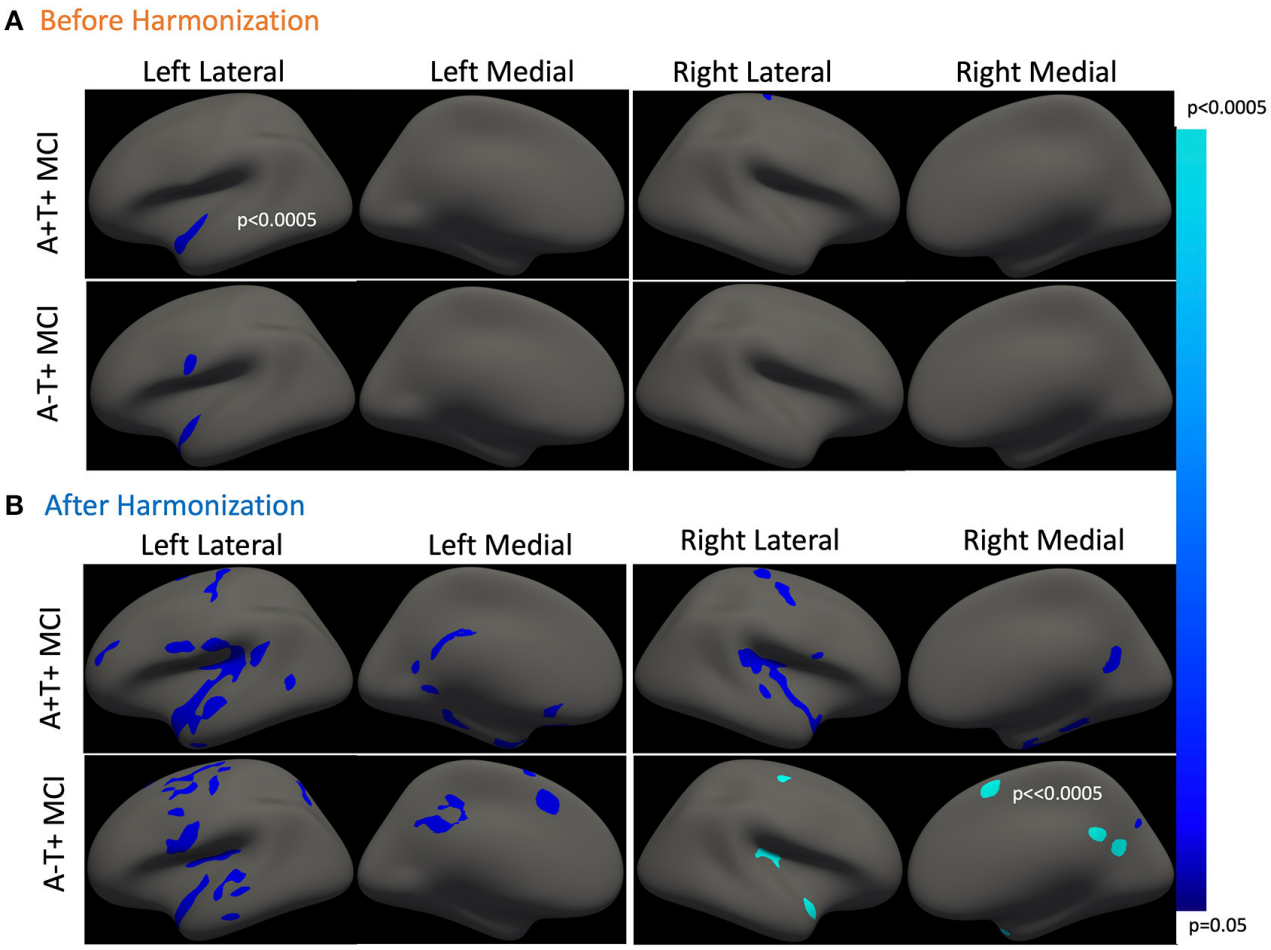
Association between cross-sectional cortical thickness and age. **(A)** This shows thinner cortical thickness with increasing age in amyloid positive and A-T+ MCI. Both groups show similar cortical atrophy in the temporal cortex. The results were lateralized to the left hemisphere. **(B)** This shows cross-sectional cortical thinning with age in the same two groups after harmonization. The cortical thinning is more bilateral and widespread in both groups. Table 1 outlined all regions or clusters identified using the Desikan Killany atlas with a cluster-wise *p* < 0.05 before and after harmonization. The average *Z*-value of all vertices in the cluster is also included in Table 1. Blue-color bar represents negative association between age and cortical thickness, i.e., higher the age, thinner the cortex, with lighter blue representing more significant *p*-values.

Overall, we did not find any group differences in cortical thickness between groups. Cross-sectionally, reduced cortical thickness was associated with older age in the left temporal cortex in the A-T+ MCI participants, as shown in Figure 1A. This observation was made on unharmonized data. As expected, no observations of significance were observed in the NC group. The p-values refer to the probability of the cluster. The average vertex-wise *Z* score for each cluster along with the cluster-wise *p*-values are reported in Table 1.

**Table 1:** Comparison of cross-sectional associations between cortical thickness and age before and after applying harmonization (with reference to Figure 1).

**Group/region**	**Before harmonization (1A) Cluster p–values (mean vertex Z)**		**After harmonization (1B) Cluster p–values (mean vertex Z)**	
**A+T+ MCI**	**Left cortex**	**Right cortex**	**Left cortex**	**Right cortex**
Superior temporal	0.0002 (−4.6)		0.0002 (-4.5)	0.0002 (-4.2)
Precentral	0.02 (−4.2)		0.0002 (-4.4)	0.0002 (-4.3)
Superior frontal	0.03 (−4.2)		0.002 (-4.2)	
Entorhinal			0.0002 (-4.3)	
Parahippocampal			0.0002 (-4.4)	
Rostral middle frontal			0.0002(-4.4)	
Isthmus Cingulate			0.0002 (-4.2)	
Middle temporal			0.0002 (-4.4)	
Inferior temporal			0.002 (-4.0)	
Precuneus			0.002 (-4.3)	0.0002 (-4.6)
Medial orbitofrontal			0.003 (-4.0)	
Lingual			0.005 (-4.4)	
Rostral anterior cingulate			0.008 (-4.5)	
Postcentral			0.02 (-4.2)	
Paracentral			0.02 (-4.2)	
Inferior parietal			0.03 (-4.1)	
Fusiform				0.002 (-4.3)
Supramarginal				0.0002 (-4.5)
**A-T+** **MCI**	**Left cortex**	**Right cortex**	**Left cortex**	**Right cortex**
Superior temporal	<0.001 (−4.2)		0.0002 (-4.3)	0.0002 (-4.5)
Precentral	0.002(−4.2)		0.0002 (-4.6)	0.0002 (-4.3)
Superior frontal	0.03 (−4.3)		0.0002 (-4.9)	0.0002 (-4.6)
Insula			0.0002 (-4.4)	
Caudal middle frontal			0.0002 (-4.2)	
Isthmus cingulate			0.0002 (-4.4)	0.001 (-4.5)
Superior parietal			0.0002 (-4.2)	
Precuneus			0.0002 (-4.1)	0.0002 (-4.1)
Middle temporal			0.006 (-4.4)	
Rostral anterior cingulate			0.02 (-4.4)	
Supramarginal				0.0002 (-4.6)
Temporal pole				0.003 (-4.2)
Lateral orbitofrontal				0.03 (-4.2)

**Figure 2:**
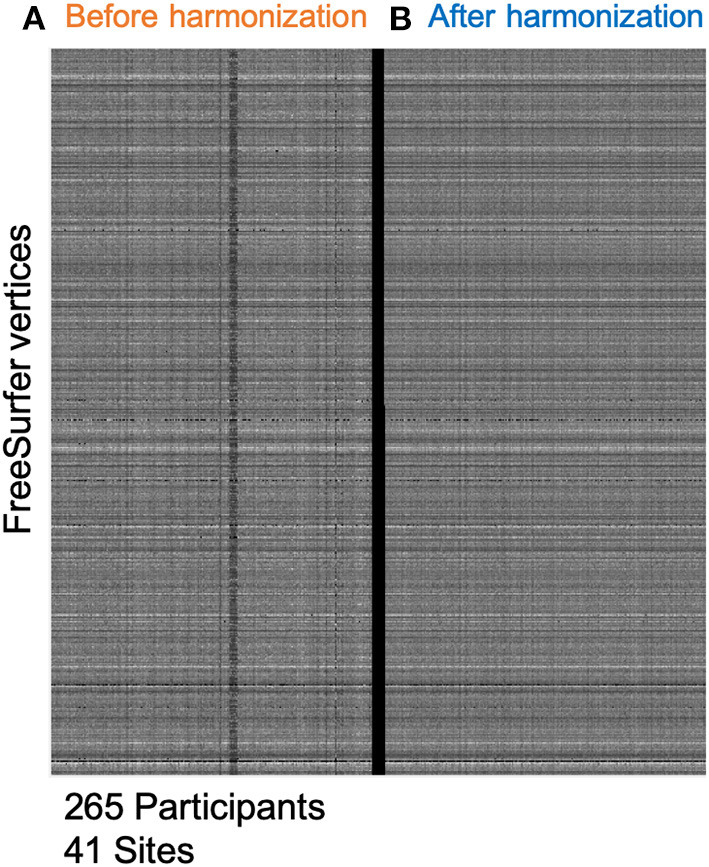
Minimizing site-effects using ComBat Harmonization. This study included 265 participants from 41 different sites. **(A)** This shows cortical thickness values on the FreeSurfer mesh. Each column represents one subject, and each row represents cortical thickness at each vertex on the FreeSurfer 3D mesh. **(B)** This shows a similar plot after applying the ComBat routine with parametric adjustments. The carpet plot of cortical thicknesses clearly shows site-specific differences along the columns before harmonization. After harmonization, the carpet plot appears smoother.

Figure 2 shows a carpet-plot of cortical thickness data in all 265 participants before and after harmonization. Each column represents a single participant, and each row represents a single vertex on the FreeSurfer surface mesh. Evidently, after harmonization, the carpet plot appears smoother with similar values for the same vertex across all participants irrespective of sites (i.e., each row). The values for the coefficient of variation in thickness measurements before harmonization and after harmonization were 13.55 ± 9.28 and 10.15 ± 7.52%, respectively; a reduction of 3.4 ± 2.38% was noted.

Figure 1B shows the cross-sectional cortical thickness and its association with age after harmonization of the cross-sectional data. Figure 1A represent the cross-sectional analyses before harmonization and Figure 1B represents the same cross-sectional analyses after harmonization. The reduction in site-related variability is clearly visible in Figure 2. After harmonization, the main effect of age on cortical thickness was bilateral and more widespread in both MCI groups. Table 1 outlines the regions from Figure 1, showing a significant main effect of age before and after harmonization. Still, no differences were observed between the groups.

**Figure 3:**
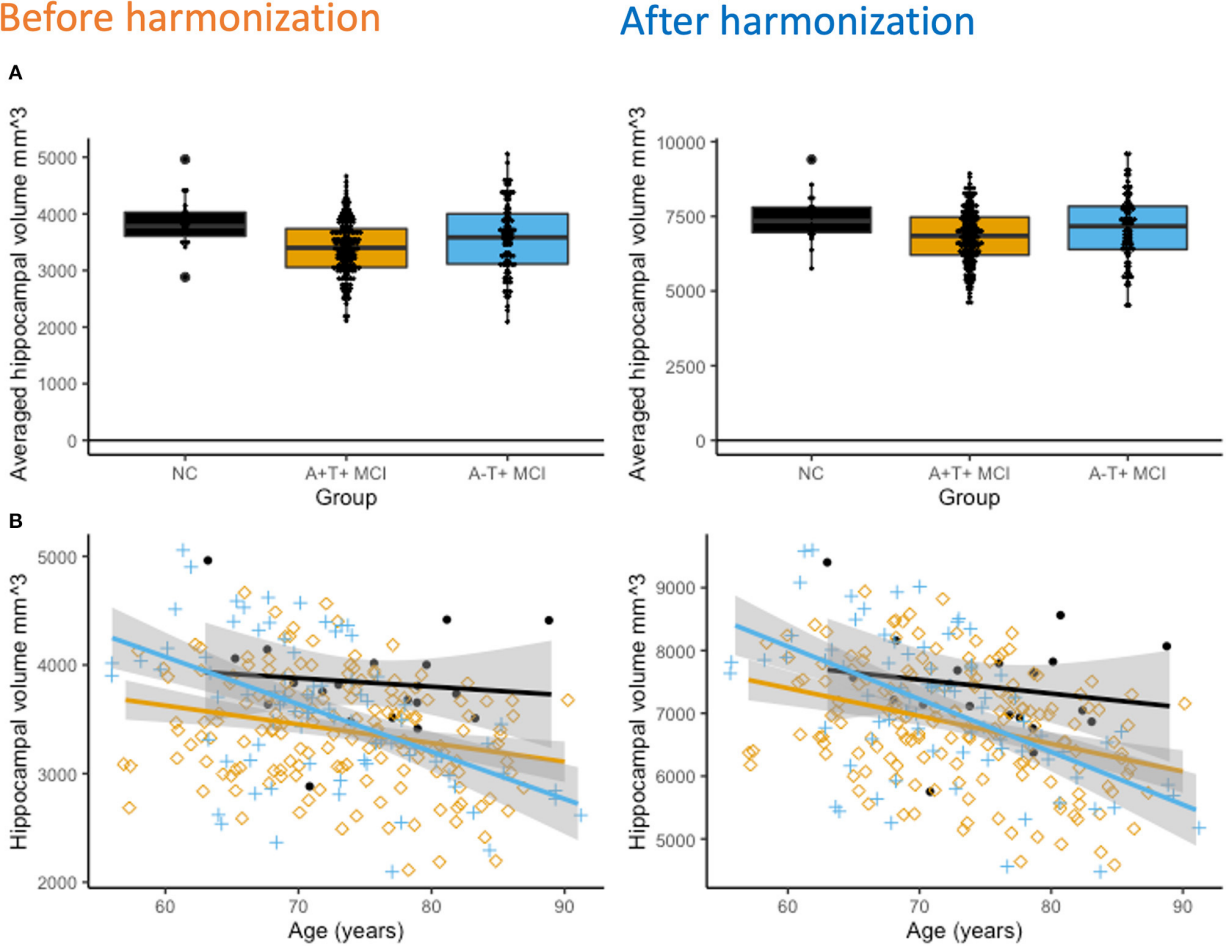
**(A)** Cross-sectional hippocampal volume comparisons. After adjusting for age, gender, and total intracranial volume (ICV), hippocampal volumes were significantly different between all three groups (*p* < 0.001 both before and after harmonization). The NC group had the highest hippocampal volume and the amyloid positive MCI group had the lowest hippocampal volume. **(B)** Interestingly, the A-T+ groups show the strongest association between decreasing hippocampal volume and increasing age, followed by the A+T+ MCI group. In the NC group, it declined the least.

Figure 3A shows hippocampal volume differences between the three groups after adjusting for age, gender, and intracranial volume (ICV). Average hippocampal volumes were the highest in the NC group, followed by the A-T+ MCI group, and it was the least in the A+T+ MCI group. Group differences were significant (*p* < < 0.001) and not affected by harmonization. Cross-sectional, age-related hippocampal atrophy was the least in controls, followed by the A+T+ MCI group, and then the A-T+ MCI group (Figure 3B). The lack of relationship between hippocampal volume and age in controls could also be due to a smaller sample size. These patterns were unchanged with harmonization. The coefficient of variations reduced by 10.30% from 16.4% to 14.7% bilaterally in the hippocampus and by 7.7% in the ICV measurements. Note that the plots are outcomes of regression analysis; hence no correlation values are shown.

### Longitudinal Comparisons

**Figure 4:**
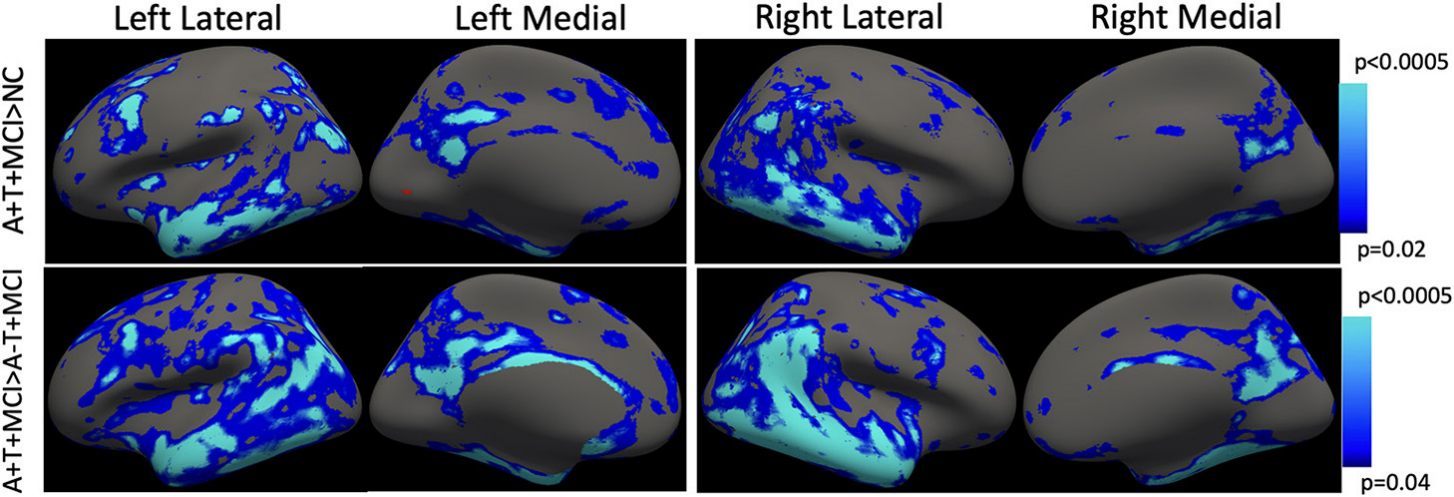
Longitudinal cortical thinning. Using the mass-univariate linear mixed-effects model proposed by FreeSurfer, MCI+ group showed a faster rate of atrophy compared with both NC and SNAP groups. The *p*-values indicate the cut-off threshold determined by the model after adjusting for multiple comparisons and separate evaluation of the two cortical surfaces. Blue-color bar represents cortical thinning in the AT+ MCI group compared with NC and compared with A-T+ MCI, with lighter blue representing more significant *p*-values. Multiple comparisons correction is achieved using false discovery rate (FDR). No significant difference was observed between NC and SNAP groups.

Longitudinal cortical atrophy differences are depicted in Figure 4. Using the advanced linear mixed-effects model in FreeSurfer tools, the A+T+ group had significantly higher rates of cortical thinning compared to the NC group bilaterally in the temporal lobe (superior, middle, and inferior temporal gyri), superior frontal gyrus, posterior cingulate, and precuneal regions, as well as anterior cingulate (*p* < 0.02). The A+T+ MCI group also showed significantly higher rates of longitudinal cortical atrophy in the temporal lobe (superior, middle, and inferior gyri), superior frontal gyrus, and the cingulate (*p* < 0.04) when compared to the SNAP group. All results were corrected for age, gender, and multiple comparisons across tests and the two cortices using False Discovery Rate (FDR). No significant difference was observed between the A-T+ MCI group and the NC group.

Compared with NC, the A+T+ MCI participants showed a significantly faster rate of atrophy in the two hippocampi (*p* = 0.005). The A-T+ MCI participants also had a significantly faster rate of hippocampal atrophy (p=0.03). No significant difference was observed between the longitudinal rates of hippocampal atrophy between A-T+ MCI and A+T+ MCI participants (*p* = 0.67).

## Discussion

This study has three observations. (1) Using a large dataset from multiple sites, this study shows that with age, the A+T+ and A-T+ MCI groups have a similar pattern of cortical thinning and hippocampal atrophy cross-sectionally, as shown in previous studies. (2) Novel in this study is the comparison of hippocampal atrophy and cortical thinning in the two groups. Longitudinally, both MCI groups showed a higher annual rate of hippocampal atrophy. No significant differences were observed in the longitudinal hippocampal atrophy rates between the two groups. (3) However, only the A+T+ group showed a significantly faster rate of cortical thinning compared with both NC and A-T+ MCI groups. (4) Harmonization greatly minimized site differences and improved the detection of group differences.

We studied a sub-group of amyloid-negative MCI participants with elevated p-tau, since it is also a signature of AD pathology (Baldeiras et al., 2018). In our study, the p-tau levels in ADNI were 50.6 ± 21.8 pg/ml in A+T+ MCI and 33 ± 9.9 pg/ml in A-T+ MCI group. The respective values were 96.6 ± 19.0 and 96.0 ± 22.1 pg/ml in ARWiBo, 89.4 ± 22.0 and 104 ± 49.7 pg/ml in EDSD, and 93.0 ± 35 and 92.9 ± 31.8 pg/ml in PharmaCog. All the values are above the cut-off for p-tau positivity. The equal distribution of the neurodegeneration marker N in the two MCI groups, the lack of longitudinal cortical thinning in our large A-T+ MCI cohort, and the presence of only hippocampal atrophy support that A-T+ MCI group with elevated p-tau levels may be reflective of PART pathology and can be confirmed only by histopathological studies (Jack et al., 2016a). In that case, this sub-group may not develop amyloid pathology (Crary et al., 2014) or become amyloid positive in subsequent years (Gordon et al., 2015; Burnham et al., 2016). The A-T+ MCI group had a higher hippocampal volume and faster rates of atrophy, while the A+T+ MCI groups had a significantly lower hippocampal volume and a slower rate of atrophy. This is in accordance with other studies showing different baseline hippocampal volumes and rates of atrophy depending on the A/T/N status (Burnham et al., 2016). The A+T+ MCI group likely showed a higher rate of atrophy earlier and hence has more severe cognitive symptoms (poorer MMSE scores) compared with the A-T+ MCI group. In a study by Holland et al., hippocampal volume was the lowest at 65 years of age in AD, and the rate of atrophy decreased indicating the process of hippocampal volume loss was prolific in the earlier decade (Holland et al., 2012). Contrarily, hippocampal volume was higher in MCI and controls, and the rate of atrophy increased after 65 years indicating that the hippocampal loss was an active process that began around 65 years of age.

No study has measured cross-sectional cortical thickness or longitudinal rates of cortical thinning in amyloid-negative, p-tau positive MCI groups. To our knowledge, this is the first study to leverage the GAAIN platform to consolidate and further harmonize datasets across multiple repositories and provide adequate subjects for a thickness-based analysis. Studies of sample size estimates suggest a sample size of at least 50 to detect robust differences in cortical thickness comparisons. Our approach provided us with 91 SNAP participants. Furthermore, we applied ComBat harmonization to reduce site-related variability, and the differences due to harmonization are evident in Figure 2. We could only identify 20 NC participants. Only the ADNI dataset had NC participants with concurrent CSF and T1 MRI data, and most were A+. Hence, all cross-sectional comparisons with the NC group were considered secondary analyses. Note that previous studies of amyloid negative MCI groups (not necessarily T+) used NCs as amyloid-negative participants with normal cognition (disregarding the status of T and sometimes also, N) or included only MCI participants (Caroli et al., 2015; Wisse et al., 2015, 2018). Our approach chose an NC group with individuals without AD-related amyloid and tau pathology, resulting in a small number of A-T-control participants.

For longitudinal analyses, no harmonization was performed since a subject-specific template is created for estimating annual rates of atrophy. With our approach, no longitudinal deficits in cortical thickness were observed in A-T+ group. Deficits were prevalent in the A+T+ group only. No longitudinal study exists that evaluates cortical thinning in the A-T+ MCI group. The lack of a longitudinal cortical atrophy signature could explain the slow progression or stable nature of these individuals, but further investigations are warranted. One issue is that scanners often undergo upgrades that may introduce variability even in longitudinal measurements, and these are not accounted for in this study.

Most studies of the MCI population are performed in a mixed MCI population irrespective of amyloid status or only in amyloid-positive MCI participants. Our findings are very much in accordance with previous studies of amyloid-positive MCI participants, showing higher atrophy compared with controls (Huijbers et al., 2015; Nosheny et al., 2015; Jang et al., 2019). Cortical thinning has been most commonly reported in the medial temporal regions, parieto-occipital regions, and the frontal regions, also known as AD-vulnerable regions. These patterns of atrophy are more conspicuous in A+ MCI participants (Becker et al., 2011; Ekman et al., 2018). Importantly, these regions also show the earliest amyloid accumulation in AD beside the hippocampus. Amyloid accumulation is known to exit even decades before AD symptoms manifest, and it is associated with greater thinning in the AD vulnerable regions (Doré et al., 2013). Amyloid is known to cause vasoconstriction, increase inflammation, and oxidative stress, all of which eventually result in neuronal injury or death (Zlokovic, 2005). It is, therefore, not surprising that amyloid status plays an important role in brain atrophy. Phosphorylated tau, p-tau_181_, is associated with axonal transport, cell signaling, microtubule stabilization, and tau release. P-tau_181_ activity is regulated by enzymes that are also partly affected by amyloid. Markers of abnormal p-tau and amyloid together increase the specificity to AD compared with either one of them, indicating that amyloid and p-tau have distinct effects on AD pathogenesis.

Whether A-T+ is a distinct pathological state still remains unknown. Even with the larger sample sizes, we achieved in our study, no cortical signatures were identified. Hippocampal atrophy was similar in A-T+ and A+T+ MCI groups.

## Conclusions

Our study shows that A-T+ MCI participants have higher hippocampal volume loss at baseline and similar longitudinal rates of atrophy as amyloid positive MCI participants. Only the amyloid-positive MCI participants additionally exhibit widespread cortical thinning longitudinally. This study included a very limited number of cognitively normal controls. Future studies are needed with a larger sample of amyloid and p-tau negative cognitively normal controls.

## Data Availability Statement

Publicly available datasets were analyzed in this study. This data can be found here: Alzheimer's Disease Neuroimaging Initiative (ADNI), https://adni.loni.usc.edu/ and The Global Alzheimer's Association Interactive Network (GAAIN), http://www.gaain.org/.

## Ethics Statement

Ethical review and approval was not required for the study on human participants in accordance with the local legislation and institutional requirements. Written informed consent to participate in this study was provided by the patient/participants.

## Author Contributions

The author confirms being the sole contributor of this work and has approved it for publication. ADNI and GAAIN provided access to existing de-identified data. They are not involved in the scientific hypothesis generation, processing, and analysis or interpretation of the work in this publication.

## Funding

This work is supported by the GAAIN Exploration to Evaluate Novel Alzheimer's Queries (GEENA-Q-19-595184) grant awarded by the Alzheimer's Association to SR. Data collection and sharing for this project were funded by the Alzheimer's Disease Neuroimaging Initiative (ADNI) (National Institutes of Health Grant U01 AG024904) and DOD ADNI (Department of Defense award number W81XWH-12-2-0012). ADNI is funded by the National Institute on Aging, the National Institute of Biomedical Imaging and Bioengineering, and through generous contributions from the following: AbbVie, Alzheimer's Association; Alzheimer's Drug Discovery Foundation, Araclon Biotech, BioClinica, Inc., Biogen, Bristol-Myers Squibb Company; CereSpir, Inc., Cogstate; Eisai Inc., Elan Pharmaceuticals, Inc., Eli Lilly and Company; EuroImmun, F. Hoffmann-La Roche Ltd and its affiliated company Genentech, Inc., Fujirebio, GE Healthcare, IXICO Ltd., Janssen Alzheimer Immunotherapy Research & Development, LLC., Johnson & Johnson Pharmaceutical Research & Development LLC., Lumosity; Lundbeck, Merck & Co., Inc., MesoScale Diagnostics, LLC., NeuroRx Research, Neurotrack Technologies, Novartis Pharmaceuticals Corporation; Pfizer Inc., Piramal Imaging, Servier, Takeda Pharmaceutical Company, and Transition Therapeutics. The Canadian Institutes of Health Research is providing funds to support ADNI clinical sites in Canada. Private sector contributions are facilitated by the Foundation for the National Institutes of Health (www.fnih.org). The grantee organization is the Northern California Institute for Research and Education, and the study is coordinated by the Alzheimer's Therapeutic Research Institute at the University of Southern California. ADNI data are disseminated by the Laboratory for Neuro Imaging at the University of Southern California. EDSD was partially supported by a grant of the Department Individual and Society Aging of the Interdisciplinary Faculty of the University of Rostock and by a grant of the Hirnliga e.V Foundation. PharmaCOG funding was received from the European Community's Seventh Framework Programme (FP7/2007–2013) for the Innovative Medicine Initiative under grant agreement number 115009. NeuGRID was originally funded by grant 283562 from the European Commission and further upgraded and developed by the Neuroinformatics Lab of IRCCS Istituto Centro San Giovanni di Dio Fatebenefratelli, Brescia Italy.

## Conflict of Interest

This study received funding from the GAAIN Exploration to Evaluate Novel Alzheimer's Queries (GEENA-Q-19-595184) grant awarded by the Alzheimer's Association to the author. The funder was involved in providing a common platform to access existing de-identified open source imaging, demographic, and biomarkers data from Alzheimer's Disease Neuroimaging Initiative, Alzheimer's Disease Repository Without Borders, European Diffusion Tensor Imaging Study on Dementia, and the Prediction of cognitive properties of new drug candidates for neurodegenerative diseases in early clinical development study. The funder was not directly involved in this specific study design, analysis, interpretation of data, the writing of this article or the decision to submit it for publication. The author declares that the research was conducted in the absence of any commercial or financial relationships that could be construed as a potential conflict of interest.

## Publisher's Note

All claims expressed in this article are solely those of the authors and do not necessarily represent those of their affiliated organizations, or those of the publisher, the editors and the reviewers. Any product that may be evaluated in this article, or claim that may be made by its manufacturer, is not guaranteed or endorsed by the publisher.
