# Supplementary material for: Cross-Sectional and Longitudinal Hippocampal Atrophy, Not Cortical Thinning, Occurs in Amyloid-Negative, p-Tau-Positive, Older Adults With Non-Amyloid Pathology and Mild Cognitive Impairment
Source: Front Neuroimaging. 2022 Jun 2;1:828767. doi: 10.3389/fnimg.2022.828767 (PMC10406207; doi:10.3389/fnimg.2022.828767)
Supplement: Supplementary file 1 [file Table_1.docx]

**Table 1:** Participant selection for the final cross-sectional and longitudinal analyses.

| **Diagnostic group** | **Initial sample with MRI and CSF at baseline/screening visit** | **Fit criteria for diagnostic group** | **Passed QC for baseline** | **Available for longitudinal analyses** | **Passed QC for longitudinal** |
| --- | --- | --- | --- | --- | --- |
| **Study: ADNI** |  |  |  |  |  |
| NC | 115 | 22 | **20** | 20 | **18** |
| A+T+ MCI | 146 | 109 | **109** | 109 | **109** |
| A-T+ MCI | 58 | 58 | **44** | 44 | **41** |
| **Study: EDSD** |  |  |  |  |  |
| NC | 0 | 0 | **0** | 0 | **0** |
| A+T+ MCI | 22 | 22 | **22** | 0 | **0** |
| A-T+ MCI | 17 | 17 | **17** | 0 | **0** |
| **Study: ArWiBo** |  |  |  |  |  |
| NC | 0 | 0 | **0** | 0 | **0** |
| A+T+ MCI | 8 | 8 | **8** | 0 | **0** |
| A-T+ MCI | 2 | 2 | **2** | 0 | **0** |
| **Study: PharmaCog** |  |  |  |  |  |
| NC | 0 | 0 | **0** | 0 | **0** |
| A+T+ MCI | 42 | 42 | **33** | 28 | **28** |
| A-T+ MCI | 29 | 29 | **29** | 20 | **20** |
|  |  |  |  |  |  |
| **Total** |  |  |  |  |  |
| NC |  |  | **20** |  | **18** |
| A+T+ MCI |  |  | **172** |  | **137** |
| A-T+ MCI |  |  | **92** |  | **61** |
